# Phylogenetic and biogeographic history of brook lampreys (*Lampetra*: Petromyzontidae) in the river basins of the Adriatic Sea based on DNA barcode data

**DOI:** 10.1002/ece3.10496

**Published:** 2023-09-04

**Authors:** Lukas Rüber, Andrea Gandolfi, Danilo Foresti, Luca Paltrinieri, Andrea Splendiani, Ole Seehausen

**Affiliations:** ^1^ Naturhistorisches Museum Bern Bern Switzerland; ^2^ Aquatic Ecology & Evolution Institute of Ecology and Evolution, University of Bern Bern Switzerland; ^3^ Conservation Genomics Research Unit Research and Innovation Centre ‐ Fondazione Edmund Mach San Michele all'Adige (TN) Italy; ^4^ Ufficio Della Caccia E Della Pesca Bellinzona Switzerland; ^5^ Liceo Cantonale Lugano Lugano Switzerland; ^6^ Dipartimento di Scienze della Vita e dell'Ambiente Università Politecnica delle Marche Ancona Italy; ^7^ Department of Fish Ecology and Evolution Eawag ‐ Swiss Federal Institute of Aquatic Science and Technology Kastanienbaum Switzerland

**Keywords:** biogeography, cytochrome oxidase I, DNA barcodes, freshwater lamprey, ichthyology

## Abstract

The Adriatic brook lamprey, *Lampetra* zanandreai Vladykov 1955, was described from northeastern Italy. Its distribution is thought to include left tributaries of the River Po and the river basins of the Adriatic Sea from the River Po to the River Isonzo/Soča in Italy, Switzerland and Slovenia. It also shows a geographically isolated distribution in the Potenza River on the Adriatic slope in Central Italy. *Lampetra* from the Neretva River system in Croatia and Bosnia and Herzegovina and the Morača River system in Montenegro that were previously identified as *L. zanandreai* were recently described as a new species *Lampetra soljani* Tutman, Freyhof, Dulčić, Glamuzina & Geiger 2017 based on morphological data and a genetic distance between the two species of roughly 2.5% in the DNA barcoding gene cytochrome oxidase I (COI). Since DNA barcodes for *L. zanandreai* are only available for one population from the upper Po River in northwestern Italy, we generated additional COI nucleotide sequence data of this species from Switzerland, northeastern and central Italy comprising near topotypic material and obtained GenBank sequences of the species from Slovenia to better assess the evolutionary history of the two brook lamprey species in the river basins of the Adriatic Sea. Our data show a low sequence divergence of <1% between *L. zanandreai* from Switzerland, northeastern and central Italy and Slovenia and the Balkan species *L. soljani*. However, members of the population previously identified as ‘*L. zanandreai*’ from northwest Italy are genetically highly divergent from those of *L. zanandreai* and likely belong to an undescribed species, *L*. sp. ‘upper Po’. The presence of a unique and highly divergent brook lamprey lineage in the upper Po River suggests that *L. zanandreai and Lampetra* sp. ‘upper Po’ may have evolved in separate paleo drainages during the formation of the modern Po Valley subsequent to marine inundations in the Pliocene.

## Graphical abstract

The genetic diversity and biogeographic history in brook lampreys (Lampetra: Petromyzontidae) in the river basins of the Adriatic Sea based was assessed based on DNA barcode data. The presence of a unique and highly divergent brook lamprey lineage in the upper Po River suggests that two lamprey species have evolved in separate paleo drainages during the formation of the modern Po Valley subsequent to marine inundations in the Pliocene.

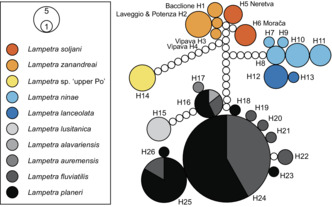

## 1 INTRODUCTION

Lampreys (Petromyzontiformes) are jawless vertebrates with an eel‐like body, a single median nostril between the eyes and seven pairs of external gill openings. Lampreys possess an oral sucking disc full of sharp, horny tooth‐like projections, which they use to attach to the substrate or to their prey. They show an antitropical distribution with the two families Geotriidae (one genus, two species) and Mordaciidae (one genus, three species) in the southern hemisphere and the family Petromyzontidae (eight genera, over 40 species) in the northern hemisphere. The evolution of their complex life cycles has attracted the interest of evolutionary biologists for decades (Cahsan et al., 2020; Espanhol et al., 2007; Hardisty, 1986; Mateus et al., 2013, 2016; Rougemont et al., 2017; Zanandrea, 1959, 1961). Shortly after spawning the adult lampreys die and their hatched larvae (ammocoete) show an extensive larval period of up to several year, which they spend buried in the sediments of brooks, feeding by filtrating food items out of the water. After metamorphosis juveniles of anadromous species migrate to the sea where they spend one to several years growing and maturing before they migrate upstream to reproduce in freshwater. However, several non‐migratory or sedentary species, that spend their entire life cycle in freshwater, are known. As adults, migratory species attach with their oral sucking disc on fish or other prey and live either as predators or as parasites by using their rough tongue‐like apparatus to rasp away flesh from their victims or to feed on blood and body fluids. In contrast, many non‐migratory species do not feed at all after metamorphosis.

Closely related lamprey species often show divergent life cycles with one anadromous and predatory/parasitic species and one or several non‐migratory and non‐predatory/non‐parasitic ‘satellite’ species probably derived from the anadromous species (Vladykov & Kott, 1979; Zanandrea, 1959). The European river lamprey, *Lampetra fluviatilis* (Linnaeus 1758), an anadromous, predatory species and the European brook lamprey, *Lampetra planeri* (Bloch 1784), a freshwater, non‐predatory species are one of the best studied models to better understand life history evolution in migratory and non‐migratory species (Cahsan et al., 2020; Espanhol et al., 2007; Mateus et al., 2011, 2013, 2016; Rougemont et al., 2016, 2017).

Recent phylogenetic studies (Lang et al., 2009; Pereira et al., 2021) have indicated that the genus *Lampetra* should be restricted to species from Europe, the Caucasus and Asia Minor. It currently comprises one migratory species, *L. fluviatilis* found in the Baltic Sea, the North Sea, the northeastern Atlantic and the western Mediterranean and adjacent freshwater habitats and eight non‐migratory freshwater species: *Lampetra planeri*, a widespread species found in western Europe; *Lampetra lanceolata* Kux & Steiner 1972 and *Lampetra ninae* Naseka, Tuniyev & Renaud 2009, restricted to the southern and western Black Sea basin, respectively; *Lampetra alavariensis* Mateus, Alves, Quintella & Almeida 2013, *Lampetra auremensis* Mateus, Alves, Quintella & Almeida 2013, *Lampetra lusitanica* Mateus, Alves, Quintella & Almeida 2013, three allopatric species with restricted distributions in Portugal; and two allopatric species confined to the river basins of the Adriatic Sea, *Lampetra zanandreai* Vladykov, 1955 and *Lampetra soljani* Tutman, Freyhof, Dulčić, Glamuzina & Geiger 2017.

*Lampetra zanandreai*, the Adriatic brook lamprey, was described based on type material from the north Italian province of Vicenza, and additional material from the river Ticino, presumably from Pavia, Italy (Vladykov, 1955). According to Zanandrea (1963), the species is found in Italy and Switzerland in left tributaries of the River Po and in river basin of the Adriatic Sea from the River Po to the River Isonzo/Soča that drains into the Gulf of Trieste. The species was also recorded from the River Vipa, a tributary of the River Isonzo/Soča in Slovenia (Povž, 1992, 1995; Zanandrea, 1963). Subsequently an isolated occurrence was reported for *L. zanandreai* from the River Potenza in the Marche region on the Adriatic slope in Central Italy (Bianco, 1992). According to literature cited in Bianco (1992), the species might historically also have occurred in the nearby river Esino. Another isolated occurrence was recorded with the discovery of *L. zanandreai* in the Neretva River basin in Croatia (Holčík & Mrakovčić, 1997) and Bosnia and Herzegovina (Tutman et al., 2009). In addition, Holčík and Šorić (2004) listed comparative material of *L. zanandreai* from the rivers Morača and Zeta (Morača River system) in Montenegro (see also Šanda et al., 2005). Interestingly, in a study focussing on the mitochondrial DNA variation of *L. zanandreai* in Italy, Caputo et al. (2009) found 0.0%–0.9% uncorrected sequence divergence in a 231 bp segment of the cytochrome *b* (cyt*b*) gene among the isolated River Potenza population in central Italy and populations from the eastern (lower) Po Plain in northern Italy, but 2.6%–3.5% uncorrected sequence divergence between these two populations and the western (upper) Po Plain populations.

In their extensive DNA barcoding study of Mediterranean freshwater fish species, Geiger et al. (2014) referred to the *Lampetra* from the upper Po in Italy (44.873 N 7.676 E) as *L. zanandreai* while for those from the Neretva Drainage in Bosnia and Herzegovina (43.053 N 17.698 E) and from the Morača River system that flows into Lake Skadar in Montenegro (42.31 N 19.199 E) as *Lampetra* sp. and not as *L. zanandreai*. These two species were distinguished by 2.5% K2P (Kimura 2‐parameter) divergence in the cytochrome oxidase I (COI) barcoding sequence and were not resolved as a monphyletic group. Based on the genetic data of Geiger et al. (2014) and additional GenBank sequences of other *Lampetra* species and novel morphological data, Tutman et al. (2017) subsequently described the *Lampetra* from the Neretva and Morača drainages as *Lampetra soljani*. Using a comprehensive sampling of *Lampetra* COI sequences, Tutman et al. (2017) showed that so‐called *L. zanandreai* from the upper Po in Italy are the sistergroup to a group comprising *L. soljani* and the two species from the Black Sea basin, *L. ninae* and *L. lanceolata*.

Preliminary analyses of DNA barcodes from *L. zanandreai* from Switzerland revealed a close relationship with *L. soljani* and large genetic differences with the *L. zanandreai* used by Geiger et al. (2014) and Tutman et al. (2017). Thus, to better understand the evolutionary history of the genus *Lampetra* in the river basins of the Adriatic Sea, we sequenced additional *L. zanandreai* from Italy, including a population from near to the type locality and one from the central Adriatic slope and analysed them together with a comprehensive sampling of *Lampetra* mined from GenBank.

## 2 MATERIALS AND METHODS

In order to assess the evolutionary history of *Lampetra* from the river basins of the Adriatic Basin, we downloaded available COI nucleotide sequences from GenBank and generated new COI sequences from eight specimens of *Lampetra zanandreai* from Switzerland (*n* = 2, River Laveggio, a tributary of the River Ticino, Po River system) and two localities from Italy (*n* = 2, River Bacchglione, Brenta River system, a locality close to the type locality of the species and; *n* = 4, River Potenza, central Italy). Total genomic DNA was extracted from muscle tissue or fin‐fold clips preserved in 100% ethanol and stored at −80°C. We used the DNeasy Blood and Tissue Kit on a QIAcube robotic workstation following the manufacturer's instructions (Qiagen). The universal COI barcoding region was amplified using the following primers combinations: FishF1 and FishR1 or FishF2 and FishR2 (Ward et al., 2005). PCR protocol and PCR conditions follow Conte‐Grand et al. (2017). PCR products were cleaned and Sanger sequenced in both directions by LGC Genomics (Berlin, Germany), using the same primer pairs that were used for the PCR amplification. Raw reads were edited and assembled into contigs using Geneious Prime v2022.0.2 (https://www.geneious.com) and individual consensus sequences together with sequences available at GenBank were aligned using MAFFT v7.017 (Katoh & Standley, 2013), as implemented in Geneious Prime. Based on previously published phylogenies of Petromyzontidae (Lang et al., 2009; Pereira et al., 2021), we chose *Eudontomyzon mariae* (GenBank accession ON097571) as outgroup.

Maximum likelihood analyses were conducted with RAxML v7.3.4 (Stamatakis, 2014) under the ‘‐f a’ setting and rapid bootstrap replicates. The optimal partition scheme was generated using PartitionFinder 1.0.1 (Lanfear et al., 2012) using initial partitions according to codon position. The setting model_selection = BIC and search = greedy was used for the PartitionFinder run (models = raxml). RAxML v7.3.4 (Stamatakis, 2014) was used to reconstruct the maximum likelihood (ML) tree implementing the GTRGAMMA model and using the option ‘‐f a’ and a rapid bootstrap analysis with 1000 pseudoreplicates.

PopART (Leigh & Bryant, 2015) was used to reconstruct a median‐joining network (Bandelt et al., 1999) using default settings (epsilon = 0). Since PopArt ignores alignment positions with missing information, seven sequences with missing data including the first or last variable position in the alignment were excluded from this analysis (Table S1). SplitsTree4 v4.18.3 (Huson & Bryant, 2006) was used to reconstruct a Neigbornet based on logDet distances. Genetic distances (*p*‐distances) within and between species were calculated in PAUP* v4.0a147 (Swofford, 2002).

## 3 RESULTS

**FIGURE 1:**
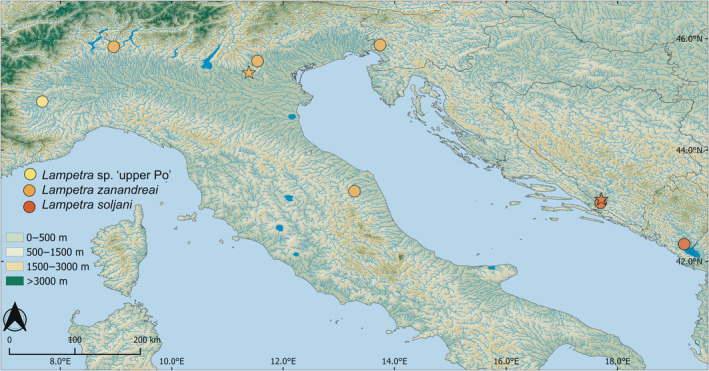
Northern Adriatic sea and surrounding areas showing the sampling location of samples used in this study: *Lampetra* sp. ‘upper Po’ (yellow), *Lampetra zanandreai* (orange) and *L. soljani* (red). Filled circles are sampling locations for specimens used in this study (GenBank or newly generated COI sequences, see Table 1 for details). Type localies of *L. zanandreai* and *L. soljani* are indicated by a star.

The final alignment used for the analyses, which included 148 *Lampetra* sequences from GenBank, eight sequences of *Lampetra zanandreai* newly generated for this study and one outgroup sequence was 651 bp long. Metadata of all *Lampetra* specimens from river basins of the Adriatic Sea and metadata of all specimens used are provided in Table 1 and Table S1, respectively and sampling locations are shown in Figure 1.

**TABLE 1:** *Lampetra* samples from the Adriatic basin used in this study, with GenBank accession numbers, locality information and co‐ordinates.

Species identity and specimen label as used in Figure 1 and Figure S1	Source	GenBank accession number	Country	River	River system	Coordinates (lat, long)
*Lampetra zanandreai* North Italy Bacchglione LR15585	This study	OR426793	Italy	Bacchglione	Brenta	45.602 N, 11.539 E
*Lampetra zanandreai* North Italy Bacchglione LR15586	This study	OR426794	Italy	Bacchglione	Brenta	45.602 N, 11.539 E
*Lampetra zanandreai* Central Italy Potenza LR15587	This study	OR426795	Italy	Potenza	Potenza	43.264 N, 13.280 E
*Lampetra zanandreai* Central Italy Potenza LR15588	This study	OR426796	Italy	Potenza	Potenza	43.264 N, 13.280 E
*Lampetra zanandreai* Central Italy Potenza LR15589	This study	OR426797	Italy	Potenza	Potenza	43.264 N, 13.280 E
*Lampetra zanandreai* Central Italy Potenza LR15590	This study	OR426798	Italy	Potenza	Potenza	43.264 N, 13.280 E
*Lampetra zanandreai* Switzerland Laveggio LR15228	This study	OR426799	Switzerland	Laveggio^a^	Po	45.857 N, 8.959 E
*Lampetra zanandreai* Switzerland Laveggio LR15230	This study	OR426800	Switzerland	Laveggio^a^	Po	45.857 N, 8.959 E
*Lampetra zanandreai* Slovenia Vipava JN027079^b^	GenBank	JN027079	Slovenia	Vipava	Isonzo/Soča	n/a^c^
*Lampetra zanandreai* Slovenia Vipava JN027080^b^	GenBank	JN027080	Slovenia	Vipava	Isonzo/Soča	n/a^c^
*Lampetra* sp. “upper Po” North Italy KJ553679^d^	GenBank	KJ553679	Italy	Po	Po	44.873 N, 7.676 E
*Lampetra* sp. “upper Po” North Italy KJ553721^d^	GenBank	KJ553721	Italy	Po	Po	44.873 N, 7.676 E
*Lampetra* sp. “upper Po” North Italy KJ553744^d^	GenBank	KJ553744	Italy	Po	Po	44.873 N, 7.676 E
*Lampetra* sp. “upper Po” North Italy KJ553930^d^	GenBank	KJ553930	Italy	Po	Po	44.873 N, 7.676 E
*Lampetra* sp. “upper Po” North Italy KJ553977^d^	GenBank	KJ553977	Italy	Po	Po	44.873 N, 7.676 E
*Lampetra* sp. “upper Po” North Italy KJ554015^d^	GenBank	KJ554015	Italy	Po	Po	44.873 N, 7.676 E
*Lampetra soljani* Bosnia and Herzegovina Neretva KJ553665	GenBank	KJ553665	Bosnia and Herzegovina	Krupa	Neretva	43.053 N, 17.698 E
*Lampetra soljani* Bosnia and Herzegovina Neretva KJ553778	GenBank	KJ553778	Bosnia and Herzegovina	Krupa	Neretva	43.053 N, 17.698 E
*Lampetra soljani* Bosnia and Herzegovina Neretva KJ553990	GenBank	KJ553990	Bosnia and Herzegovina	Krupa	Neretva	43.053 N, 17.698 E
*Lampetra soljani* Montenegro Morača KJ553756	GenBank	KJ553756	Montenegro	Morača	Morača	42.310 N, 19.199 E
*Lampetra soljani* Montenegro Morača KJ553819	GenBank	KJ553819	Montenegro	Morača	Morača	42.310 N, 19.199 E
*Lampetra soljani* Montenegro Morača KJ553874	GenBank	KJ553874	Montenegro	Morača	Morača	42.310 N, 19.199 E
*Lampetra soljani* Montenegro Morača KJ554074	GenBank	KJ554074	Montenegro	Morača	Morača	42.310 N, 19.199 E

*Note*: A table of all samples used in this study is provided in Table S1.

^a^ The river Laveggio is a tributary of Lago di Lugano, which is connected to Laggo Maggiore. The outflow of Laggo Maggiore is the river Ticino, a tributary of the Po.

^b^ The country of origin of the two *Lampetra zanandreai* with GenBank accession numbers JN027079 and JN027080 is given as Italy. However, their catalogue number STL 1252.01 (Tissue Collection of Saint Louis University, St. Louis, Missouri, USA) corresponds to *Lampetra zanandreai* collected in the River Vipava, Slovenia (Isonzo/Soča river basin). One of these two specimens was also used by Lang et al. (2009); Nicholas J. Lang, personal communication, September 23, 2022.

^c^ Approximated for map in Figure 4 as 45.890 N, 13.740 E.

^d^ As/organism = “Lethenteron zanandreai” in GenBank.

**FIGURE 2:**
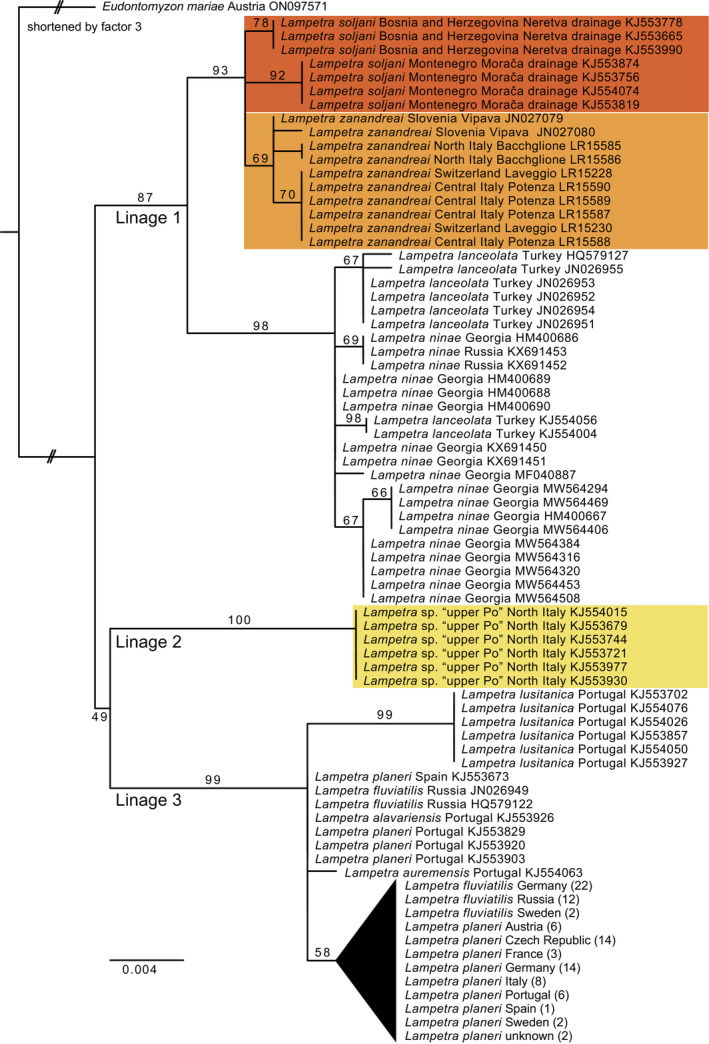
Rooted phylogram of the maximum likelihood analysis (−ln*L* = −1238.52709) of the barcoding portion of the COI gene for the genus *Lampetra*. A subclade of Lineage 3 containing 92 individuals of *L. fluviatilis* and *L. planeri* was collapsed for clarity. The uncollapsed ML tree is shown in Figure S1.

According to the results of PartitionFinder, the RAxML analyses were conducted with three partitions by codon position and the GTR + G model for each partition. The resulting ML phylogram is shown in Figure 2. Three major lineages can be identified within *Lampetra*. Lineage 1 consists of *L. soljani*, *L. zanadreai*, *L. lanceolata* and *L. ninae* with the latter two Black Sea species identified as sistergroup to the two Adriatic species. Lineage 2 consists of *L. zanandreai* from the upper Po used by Geiger et al. (2014) and Tutman et al. (2017). Because they did not cluster with the remaining *L. zanandreai* from Switzerland, Italy (including samples close to the type locality) and Slovenia, we hereafter refer to this lineage as *Lampetra* sp. ‘upper Po’. And finally, Lineage 3 consists of the remaining *Lampetra* species, the migratory *L. fluviatilis* and the widespread non‐migratory *L. planeri* as well as the three non‐migratory species endemic to Portugal, *L. auremensis*, *L. alavariensis* and *L. lusitanica*. The three major lineages were resolved as a polytomy.

**FIGURE 3:**
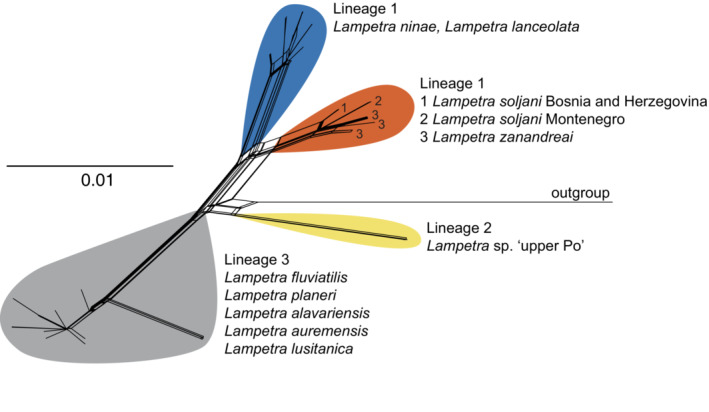
Neigbornet of the barcoding portion of the COI gene for the genus *Lampetra* with major lineages shown in different colours.

**FIGURE 4:**
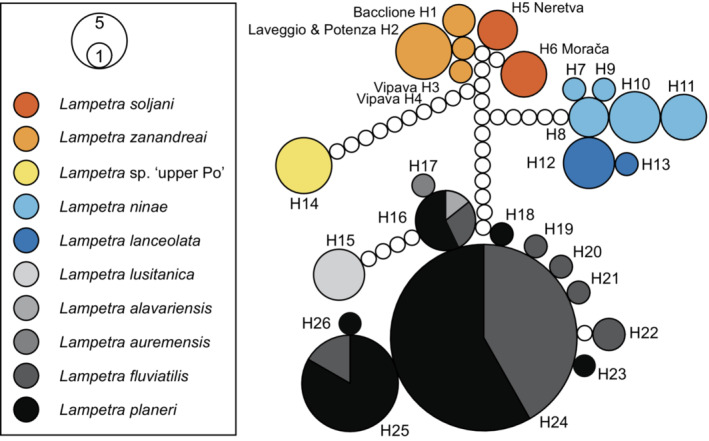
Results of the median‐joining network analyses of 26 unique haplotypes representing 149 *Lampetra* COI sequences. Haplotypes are drawn to scale, and each contact point between circles represent one mutational step and unfilled circles symbolize hypothetical, unobserved haplotypes.

Figure 3 shows the neigbornet tree and a summary of the intra‐ and interspecific *p*‐distances is provided in Table 2. Between *L. zanandreai* plus *L. soljani* and *L*. sp. ‘upper Po’ we observed between 2.15% and 2.46% uncorrected sequence divergence. Among the 149 sequences used for the analysis, 26 unique haplotypes were identified (Table S1) and the resulting median‐joining network is shown in Figure 4.

**TABLE 2:** Average and range of pairwise distances (absolute and *p*‐distances in %) among different *Lampetra* lineages based on 651 bp of the COI gene.

	*Lampetra zanandreai*, *L. soljani*	*Lampetra ninae*, *L. lanceolata*	*Lampetra* sp. “upper Po”	*Lampetra fluviatilis*, *L. planeri* (1)
*Lampetra zanandreai*, *L. soljani* (*n* = 17)	2.4 (0–4)			
	0.39% (0.00–0.61)			
*Lampetra ninae*, *L. lanceolata* (*n* = 26)	9.7 (7–11)	1.7 (0–4)		
	1.49% (1.07–1.69)	0.26% (0.00–0.61)		
*Lampetra* sp. “upper Po” (*n* = 6)	15.6 (14–16)	15.7 (13–17)	0 (0)	
	2.40% (2.15–2.46)	2.41% (2.00–2.61)	0.00%	
*Lampetra fluviatilis*, *L. planeri* ^a^ (*n* = 107)	15.0 (13–18)	15.2 (12–19)	15.4 (14–18)	1.3 (0–8)
	2.30% (2.00–2.76)	2.33% (1.84–2.92)	2.37% (2.15–2.76)	0.20% (0.00–1.22)

*Note*: Absolute distances are shown above *p*‐distance and values within a lineage are highlighted in grey.

^a^ Also includes *L. alavariensis*, *L. auremensis*, *L. lusitanica*.

## 4 DISCUSSION

### 4.1 Sequence divergence and DNA barcodes in *Lampetra*

Up until now, two species of *Lampetra* have been reported from the Adriatic basin, *Lampetra zanandreai* and *Lampetra soljani*, with the exception of a single specimen of *Lampetra fluviatilis* recorded from the Adriatic coast by Bianco and Muciaccia (1982) and the occurrence of *Lampetra planeri* in the River Pescara in central Italy (Cahsan et al., 2020; Zanandrea, 1963). Our study has shown that the *L. zanandreai* from the upper Po (= *L*. sp. “upper Po”), a few km upstream from Turin, used in previous studies (Geiger et al., 2014; Pereira et al., 2021; Tutman et al., 2017) is genetically very divergent from the true *L. zanandreai* from Switzerland, the lower Po Plain in Italy, the Potenza River in Central Italy, and the River Vipava, Isonzo/Soča River drainage in Slovenia. Our genetic analysis recovered a well‐supported clade consisting of the two Adriatic species, *L. zanandreai* and *L. soljani*, and the two Black Sea species (Figure 4) and genetic distances further showed that the closely related sister species, *L. zanandreai* and *L. soljani*, are separated from *L*. sp. ‘upper Po’ by 2.15%–2.46% uncorrected sequence divergence (Table 2). The results of our phylogenetic analyses and the observed genetic differences between *L. zanandreai* and *L*. sp. “upper Po” seem to support the presence of a hitherto unrecognized *Lampetra* species in the upper Po and confirm earlier reports of marked genetic differences between *L. zanandreai* populations from the upper Po (Villafranca Piemonte and Valenza, NW Italy) and three populations from the Brenta, one population from the River Sile (NE Italy) and one population from the River Potenza (Central Italy) by Caputo et al. (2009). Additional sampling is currently underway to establish the distribution of *L*. sp. ‘upper Po’ and to collect adult specimens for species description.

### 4.2 Biogeography of *Lampetra*

According to Pereira et al. (2021; their figure 4, but not their table S1), *Lampetra* and *Eudontomyzon* diverged roughly 2.9 million years ago (M.Y.B.P.) (95% highest posterior density (HPD) interval 2.3–3.7), while the age of the most recent common ancestor (MRCA) of *Lampetra* was dated at 1.8 M.Y.B.P. (95% HPD 1.4–2.4). *Lampetra* sp. ‘upper Po’ (as *L. zanandreai* in Pereira et al. (2021)) diverged from *L. soljani*, *L. lanceolata* and *L. ninae* 1.7 M.Y.B.P. (95% HPD 1.0–1.9) and the age of the MRCA of the later three species was dated at 0.8 M.Y.B.P. (95% HPD 0.5–1.2). The early evolutionary history of *Lampetra* in the Adriatic basin thus happened during the Plio‐Pleistocene.

**FIGURE 5:**
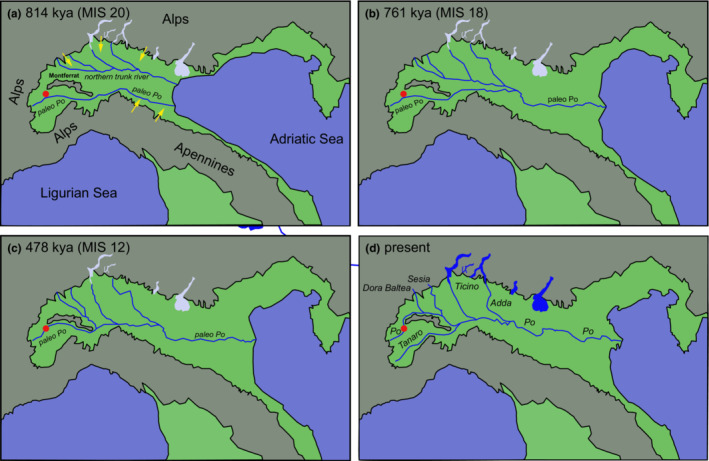
Inferred paleogeographic and paleodrainage changes in the Po Valley and the northern Adriatic Sea area based on Garzanti et al. (2011; their figures 11 and 12) for (a) Marine Isotope Stage (MIS) 20 roughly 814 kya; (b) MIS 18, roughly 761 kya; (c) MIS 12, roughly 478 kya; and (d) present.

The Po Valley, or Padanian Plain, in northern Italy is not only formed by the Po River Basin but also by adjacent lowland areas between the southern margin of the Alps and the northern margin of the Apennines. Together with the Adriatic, it is part of a tectonic foreland basin situated south of the Central, Southern and Dinaric Alps. In the Pliocene, after the Messinian salinity crisis, large parts of the Po Valley were inundated by the sea (Amadori et al., 2018; Garzanti et al., 2011; Muttoni et al., 2003; Winterberg & Willett, 2019). In the Early Pleistocene, the marine incursion became gradually smaller due to increased sedimentation of paleo rivers draining the Alps and the Apennines filling up the Po Valley and hence pushing the marine incursion to the east, resulting in a temporal west‐to‐east progression from marine to deltaic and finally to fluvial sedimentation in the Po Valley (Garzanti et al., 2011). By the Middle to Late Pleistocene, the eastward advance of the deltaic system had progressed significantly, and the marine transgression was restricted to the easternmost part of the Po Valley (Garzanti et al., 2011). This progressive basin filling resulted from the interaction among tectonic processes and the effects of Pleistocene climate cycles on Alpine glaciation accelerating erosion and sediment supply due to the waxing and waning of glaciers (Bruno et al., 2021; Garzanti et al., 2011; Muttoni et al., 2003). Initially, the exposed plain was drained by two major parallel running rivers, a northern trunk river, referred to as paleo Dora by Forno and Gianotti (2021), supplied by rivers draining the southern slopes of the Central Alps and the paleo Po supplied by rivers draining the eastern slopes of the Western Alps, the northern slopes of the Ligurian Alps and parts of the Apennines. The paleo Po was running south of the Montferrat hills, its westernmost extension being referred to as Turin Hill, and along the Apennines (Figure 5a). Increased alluvial deposits from the surrounding mountains lead to major drainage rearrangements, displacing the northern trunk river southwards and the paleo Po northward until they eventually merged (Figure 5b,c; Garzanti et al., 2011). A final major shift in drainage pattern occurred at the end of the Pleistocene (Figure 5d). Due to Montferrat uplift, the paleo Po shifted northward assuming the present course of the Po River, running north of the Montferrat hills (Forno & Gianotti, 2021; Garzanti et al., 2011; Vezzoli et al., 2010).

The reorganization of the drainages in the Po valley in the Plio‐Pleistocene likely had a major impact on the evolution and distribution of *Lampetra* in the Adriatic basin. *Lampetra* sp. ‘upper Po’ seems to be confined to the upper Po, and *L. zanandreai* is more widespread throughout the lower Po River system and adjacent river systems to the north. Further studies are needed to establish whether *L. zanandreai* and *L*. sp. ‘upper Po’ are strictly allopatric or if some areas of sympatry exist. Increased sampling together with population genomic studies, especially in the Upper Po, might allow us to test if *Lampetra* sp. ‘upper Po’ evolved in the paleo Po when it was still isolated from the northern trunk river.

River drainage evolution and connectivity throughout the Pleistocene in the Adriatic basin was further influenced by global sea level fluctuations linked to glacial cycles. During low sea levels, 100–120 m below current sea level, the Northern and parts of the Central Adriatic Sea floor were emerged. During these periods of low sea level, rivers draining the Adriatic slopes formed larger paleo rivers draining the exposed seafloor and thus connecting previously isolated river systems across the northern Adriatic basin (see figure 1 in Bianco (1992)). The possible importance of Pleistocene low sea levels resulting in an extended Po basin for *Lampetra* distribution was recognized by Bianco (1992) and could explain the low genetic divergence between *L. zanandreai* and *L. soljani*.

The sistergroup relationship of the two Adriatic species, *L. zanandreai* and *L. soljani*, with the two Black Sea species, *L. lanceolata* and *L. ninae*, is further suggestive of a very recent vicariant or dispersal event between these different areas. The lack of a migratory species in this *Lampetra* lineage comprising *L. zanandreai*, *L. soljani*, *L. lanceolata* and *L. ninae*, further challenges our current understanding on the evolution of non‐migratory lamprey species as satellite species from their putative migratory ancestors (Vladykov & Kott, 1979; Zanandrea, 1959). However, a migratory parasitic *Lampetra* of uncertain taxonomic status was recently found in the Sea of Azov (Kottelat et al., 2005; Kottelat & Freyhof, 2007; Naseka & Diripasko, 2008), from which the non‐migratory Adriatic and Black Sea *Lampetra* might have derived. Unfortunately, thus far no material for genetic work has become available from this taxon, which would help us to better understand the evolution of non‐migratory brook lampreys in the river basins of the Black Sea and the Adriatic Sea.

## AUTHOR CONTRIBUTIONS

**Lukas Rüber:** Conceptualization (equal); data curation (lead); formal analysis (lead); funding acquisition (lead); investigation (lead); methodology (lead); resources (equal); visualization (lead); writing – original draft (lead); writing – review and editing (equal). **Andrea Gandolfi:** Conceptualization (equal); funding acquisition (supporting); resources (equal); writing – review and editing (equal). **Danilo Foresti:** Conceptualization (equal); funding acquisition (supporting); resources (equal); writing – review and editing (equal). **Luca Paltrinieri:** Conceptualization (equal); funding acquisition (supporting); resources (equal); writing – review and editing (equal). **Andrea Splendiani:** Conceptualization (equal); funding acquisition (supporting); resources (equal); writing – review and editing (equal). **Ole Seehausen:** Conceptualization (equal); funding acquisition (supporting); resources (equal); writing – review and editing (equal).

## CONFLICT OF INTEREST STATEMENT

None declared.

## Supporting information


Figure S1

Table S1

## Data Availability

Data supporting the study are available in the main text or the supplementary information. Newly generated COI sequences have been deposited in the NCBI Nucleotide Archive, accession numbers OR426793–OR426800. Alignment and tree file are available on Zenodo (https://zenodo.org/record/8239010).
