# Supplementary figures and images for: Phylogenetic and biogeographic history of brook lampreys (Lampetra: Petromyzontidae) in the river basins of the Adriatic Sea based on DNA barcode data
Source: Ecol Evol. 2023 Sep 4;13(9):e10496. doi: 10.1002/ece3.10496 (PMC10477476; doi:10.1002/ece3.10496)

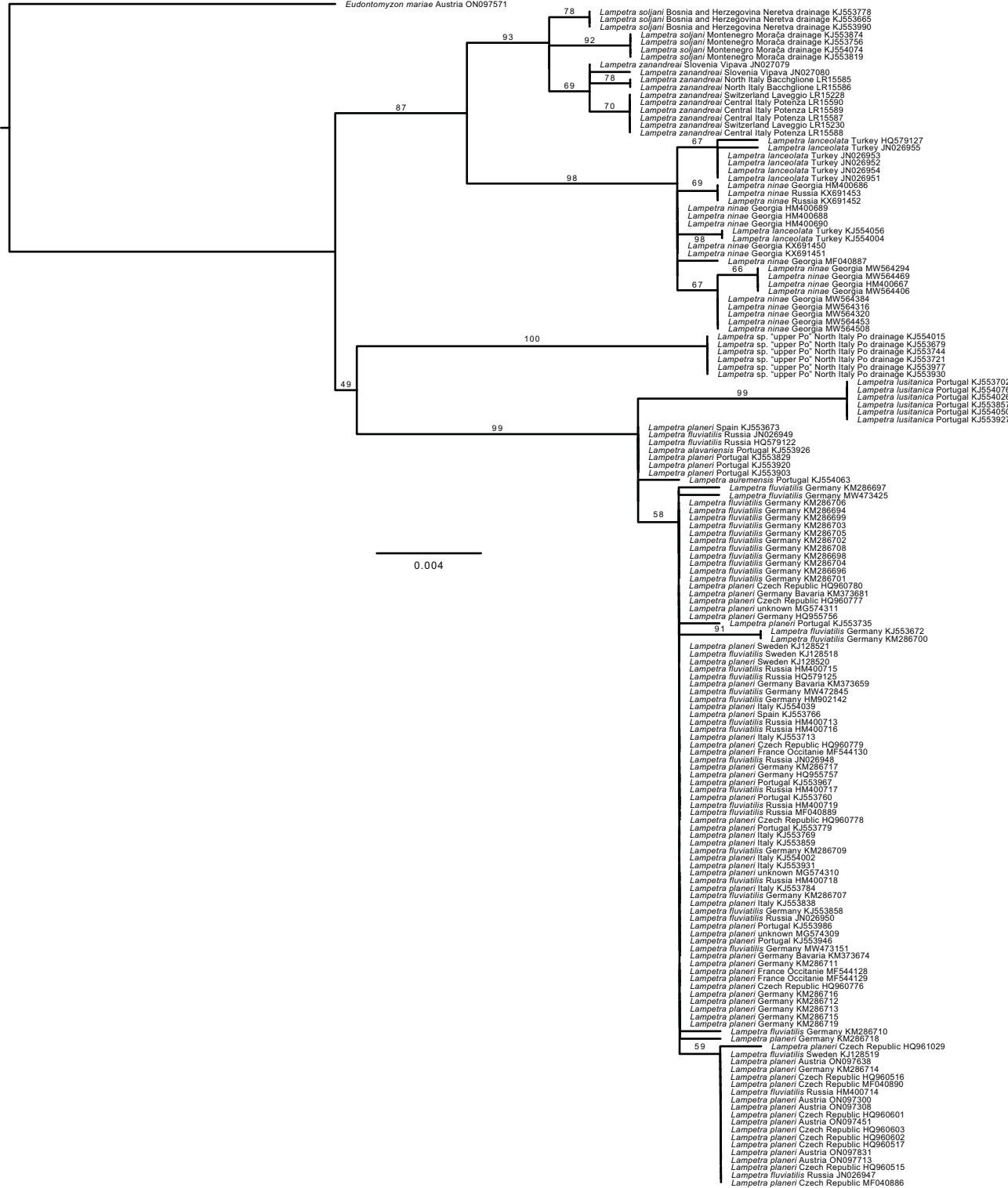

Supplement: Supplementary file 1 — Figure S1 [file ECE3-13-e10496-s002.pdf]
